# Synthesis, characterization, and evaluation of a novel inhibitor of WNT/β-catenin signaling pathway

**DOI:** 10.1186/1476-4598-12-116

**Published:** 2013-10-07

**Authors:** Zhao Yan, Zhongling Zhu, Jinghui Wang, Jian Sun, Yihui Chen, Guang Yang, Wenting Chen, Yuheng Deng

**Affiliations:** 1 Tianjin Medical University Cancer Institute and Hospital, National Clinical Research Center of Cancer, Tianjin 300060, P. R. China; 2 Key Laboratory of Cancer Prevention and Therapy, Tianjin 300060, P. R. China; 3 Beijing Laviana Pharmatech Co., Ltd, Beijing 102206, P. R. China; 4 Department of Chemistry, Capital Normal University, Beijing 100048, P. R. China

**Keywords:** β-catenin, Cell proliferation, Inhibitor, Tumorigenesis, Wnt signaling pathway

## Abstract

**Background:**

Wnt/β-catenin signaling is a highly conserved pathway in organism evolution and is important in many biological processes. Overactivation of Wnt/β-catenin signaling is closely related to tumor development and progression. To identify potent small molecules that can fight aberrant Wnt/β-catenin-mediated cancer, we synthesized a novel pyrazoline derivative (N-(4-hydroxybenzyl)-1,3,4-triphenyl-4,5-dihydro-1*H*-pyrazole-5-carboxamide, BHX) to block Wnt signaling, and determined the absolute configuration of its precursor (ethyl 1,3,4-triphenyl-4,5-dihydro-1*H*-pyrazole-5-carboxylate). We then evaluated the inhibitory effect of BHX *in vitro* and *in vivo*.

**Results:**

Cell proliferation was assessed in three human cancer cell lines (A549, HT29, and MGC803) in the presence and absence of BHX using MTS assays. BHX effectively inhibited A549, HT29, and MGC803 cell proliferation with IC_50_ of 5.43 ± 1.99, 6.95 ± 0.24, and 7.62 ± 1.31 μM, respectively. BHX significantly induced apoptosis and G1 phase arrest in A549 and MGC803 cells. The β-catenin protein level was markedly reduced in A549 and MGC803 cells under BHX treatment. The inhibitory effect of BHX *in vivo* was investigated using a mouse xenograft model. A549 xenograft growth was suppressed by 50.96% in nude mice treated continuously with 100 mg/kg BHX for 21 d. Weight remained almost unchanged, which indicates the low toxicity of the compound.

**Conclusions:**

Our data suggest that BHX is a new drug candidate for cancer treatment because of its potent effect on the Wnt/β-catenin pathway and low toxicity.

## Background

The Wnt signaling pathway is a network of proteins that are important in embryogenesis and cancer [1-4]. Wnt ligands trigger at least three different intracellular signaling cascades: the canonical Wnt pathway, which results in transcriptional regulation of target genes via β-catenin; the planar cell polarity pathway, which activates the small GTPases Rho and Rac; and the Wnt-dependent calcium/protein kinase C pathway [5]. Among these Wnt signaling pathways, the canonical Wnt/β-catenin signaling pathway is the most studied [6]. Under unstimulated conditions, β-catenin is phosphorylated by a destruction complex formed by proteins that include axin, adenomatous polyposis coli (APC), and glycogen synthase kinase-3 (GSK-3). The phosphorylated β-catenin becomes ubiquitylated and is targeted for degradation by proteasome. Following Wnt binding to transmembrane receptor Frizzled and low-density lipoprotein receptor-related protein 5/6, the destruction complex is inhibited, thereby terminating the phosphorylation of β-catenin by GSK-3. Unphosphorylated β-catenin accumulates in the cytoplasm and subsequently translocates to the nucleus where it binds to transcription factors, such as those belonging to the T cell-specific transcription factor/lymphoid enhancer-binding factor (TCF/LEF) family, and activates transcription [7].

Under pathological conditions, β-catenin escapes degradation and cells retain unregulated activation of canonical Wnt signaling caused by mutations in APC, axin, or β-catenin [8]. Activation of the Wnt/β-catenin signaling pathway is important in human tumorigenesis, including colorectal cancer [9], head and neck carcinoma [10], gastric cancer [11], melanoma [12], leukemia [13], and lung cancer [14]. The Wnt/β-catenin signaling cascade has become a major focus in cancer research. Aberrant Wnt signaling is characterized by the cytoplasmic accumulation of β-catenin and its subsequent nuclear translocation and activity [15], which suggests that β-catenin may be a potential target for drug discovery. Although several small-molecule modulators of Wnt/β-catenin signaling have been identified, none of them have been tested in clinical trials [16].

The pyrazoline family has attracted attention because of the biological activity, such as anti-inflammatory activity, of its members. Certain compounds containing the pyrazoline core also show antidepressant activity [17-19] and act as multidrug resistance modulators in tumor cells [20,21]. In this study, we synthesized a novel low molecular weight pyrazoline derivative, (4S,5R)-N-(4-hydroxybenzyl)-1,3,4-triphenyl-4,5-dihydro-1*H*-pyrazole-5-carboxamid (BHX), which acts as a Wnt/β-catenin signaling inhibitor. The derivative was biologically evaluated *in vitro* using TOPflash reporter assay (Additional file 1: Figure S1). We further evaluated the anticancer activity of BHX *in vitro* and *in vivo*. BHX may be an attractive chemotherapeutic agent because of its potent effect on the Wnt/β-catenin signaling pathway and low toxicity.

## Results

### Synthesis of BHX

**Figure 1:**
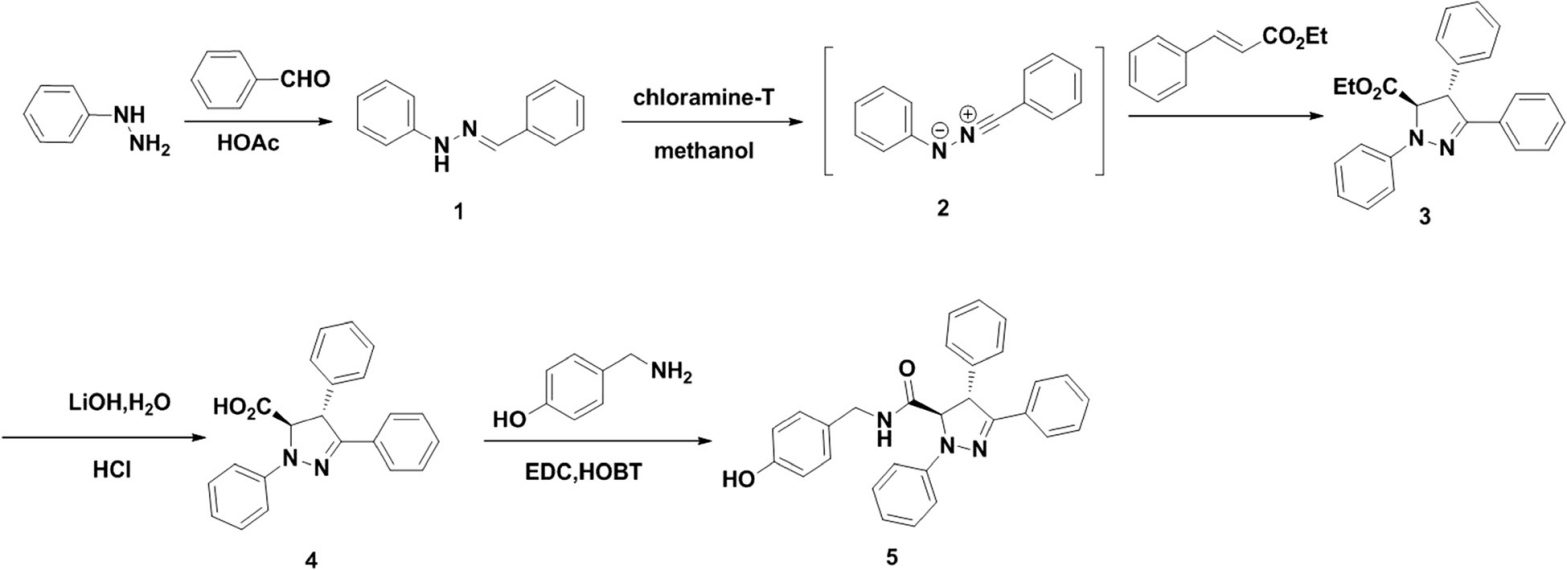
**Scheme for BHX synthesis.** 1: (*E*)-1-Benzylidene-2-phenylhydrazine; 2: diphenyl nitrilimine; 3: ethyl 1,3,4-triphenyl-4,5-dihydro-1*H*-pyrazole-5-carboxylate; 4: 1,3,4-triphenyl-4,5-dihydro-1*H*-pyrazole-5-carboxylic acid; and 5: N-(4-hydroxybenzyl)-1,3,4-triphenyl-4,5-dihydro-1*H*-pyrazole-5-carboxamide (BHX).

**Figure 2:**
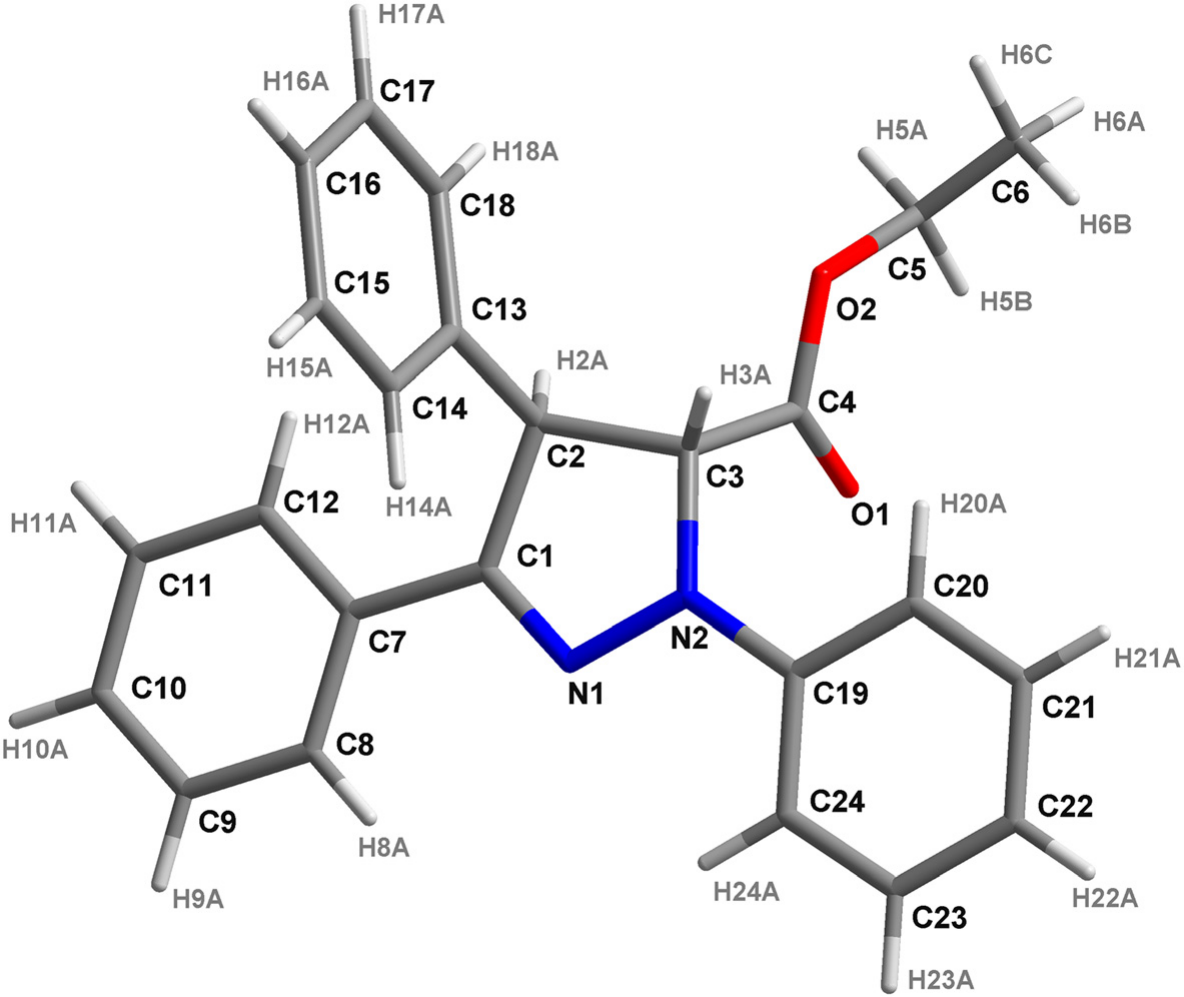
**Crystal structure of compound 3.** To confirm the regiochemistry of the cycloaddition for Δ2-pyrazoline **3**, a single crystal for compound 3 was obtained. The absolute configuration was determined using X-ray structural analysis.

The 1,3-dipolar cycloaddition reaction using nitrilimines is a well-known process [22-28]. This synthesis usually generates the corresponding pyrazolines as a mixture of regioisomers [29-31], but no direct evidence is available to reveal which regioisomers are obtained in this reaction. We prepared nitrilimine **3** by dehydrogenation of aldehyde hydrazine **1** with chloramine-T [32]. In the presence of ethyl cinnamate as dipolarophile, *in situ*-generated diphenyl nitrilimine **2** yielded the pyrazoline. After hydrolysis of compound 4 and subsequent coupling, we obtained our target compound, BHX (Figure 1). To confirm the regiochemistry of the cycloaddition for ∆2-pyrazoline **3**, ^1^H, ^13^C-HMQC and ^1^H, ^13^C-HMBC experiments were performed. We also obtained a single crystal for compound 3 (Figure 2) to determine the absolute configuration of the cycloadduct by X-ray structural analysis, which was consistent with the results of spectral analysis.

### BHX inhibits cancer cell proliferation

**Figure 3:**
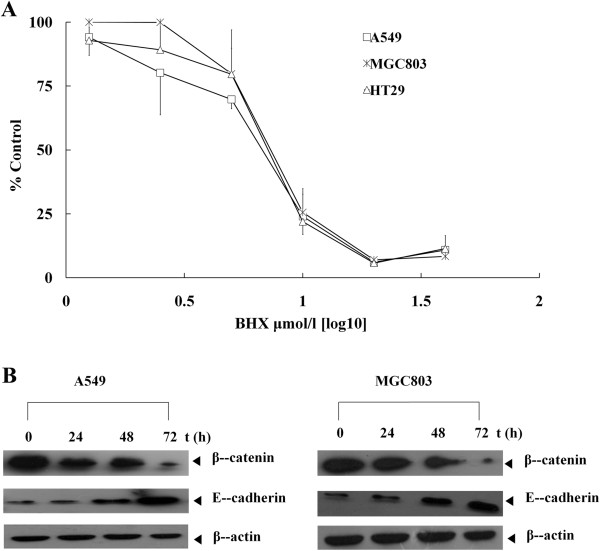
**BHX inhibits cancer cell proliferation. (A)** A549, MGC803, and HT29 cells were treated with BHX at various concentrations, and the response was determined using MTS assay. Data are presented as mean ± SD from three independent experiments. **(B)** A549 and MGC803 cells were treated with 5 μmol/L BHX for 24 h to 72 h. The β-catenin and E-cadherin levels were measured by western blot assay.

To characterize the *in vitro* activity of BHX, we used three kinds of cell lines with an overactivated Wnt signaling pathway. For each cell line, the IC_50_ was determined using MTS assay after 3 d of continuous exposure to BHX. BHX effectively inhibited human lung cancer cell line (A549), human colon cancer cell line (HT29), and human gastric cancer cell line (MGC803) cell proliferation in a dose-dependent manner, and their IC_50_ values were 5.43 ± 1.99, 6.95 ± 0.4, and 7.62 ± 1.31 μM, respectively (Figure 3A). BHX showed lower inhibitory effects on normal epithelial cells (MCF-10A) (Additional file 2: Figure S2). Changes in β-catenin protein levels were determined by western blot analysis. Introduction of BHX decreased β-catenin protein levels in either A549 or MGC803 cells in a time-dependent manner.

β-Catenin is also an essential component of the cell–cell adhesion complex by binding with E-cadherin. Wnt signaling reportedly regulates E-cadherin expression [33]. Thus, repression of E-cadherin expression by Slug/Snail or TCF/β-catenin complex reduces cell–cell adhesion. To demonstrate the effect of BHX on E-cadherin expression, E-cadherin levels were determined by western blot assay. We found that BHX induced the increase in E-cadherin protein levels (Figure 3B).

### BHX causes cell apoptosis and G1 arrest

**Figure 4:**
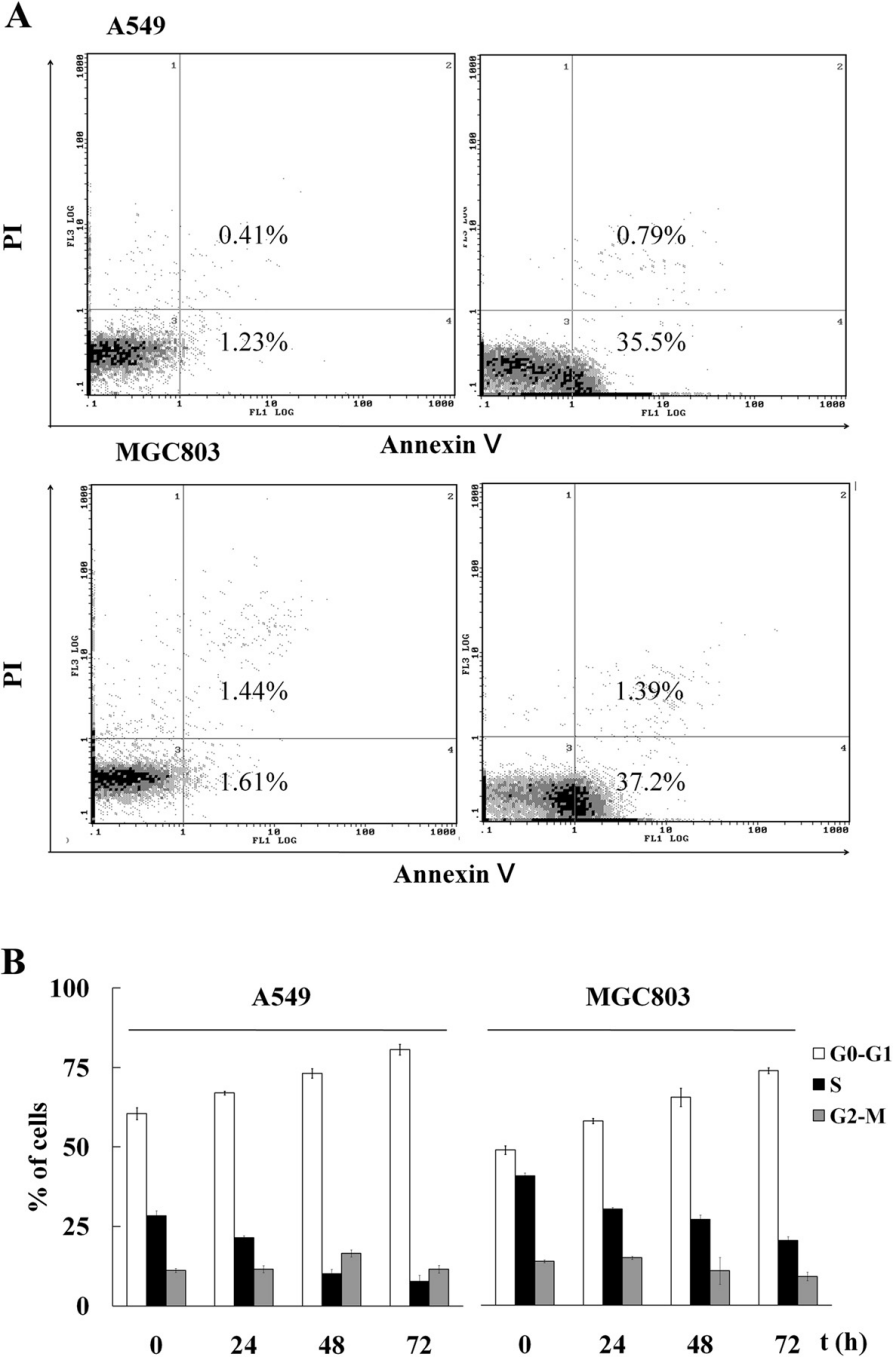
**BHX causes cell apoptosis and G1 arrest. (A)** A549 and MGC803 cells were treated with 5 μmol/L BHX for 72 h. Cell apoptosis was measured using Annexin V-FITC/PI staining. **(B)** A549 and MGC803 cells were treated with 5 μmol/L BHX for 24 h to 72 h. The percentage of cells in each cell cycle phase was determined by quantitation of DNA content by PI staining and flow cytometry. Data are presented as mean ± SD from three independent experiments.

To study BHX-induced growth inhibition, we evaluated the ability of BHX to induce A549 and MGC803 cell death by apoptosis. After treatment with 5 μmol/L BHX for 3 d, A549 and MGC803 cells displayed a significantly higher early apoptosis rate than those treated with the vehicle (*P* < 0.05). The early apoptosis rate of the BHX-treated A549 cells (35.5%) was significantly higher than that of the vehicle-treated cells (1.23%). Similarly, the early apoptosis rate of the BHX-treated MGC803 cells (37.2%) was also significantly higher than that of the vehicle-treated cells (1.61%). However, BHX-induced late apoptosis was not observed in A549 and MGC803 cells (Figure 4A). Thus, BHX possibly induced growth inhibition through an apoptosis-dependent mechanism in selected cancer cells. To study the mechanism by which BHX inhibited cancer cell growth, we also evaluated the changes in cell cycle progression using flow cytometry. BHX induced G1 arrest in A549 and MGC803 cell lines. This BHX-associated G1 arrest was time-dependent (Figure 4B).

### BHX inhibits lung cell growth *in vivo*

**Figure 5:**
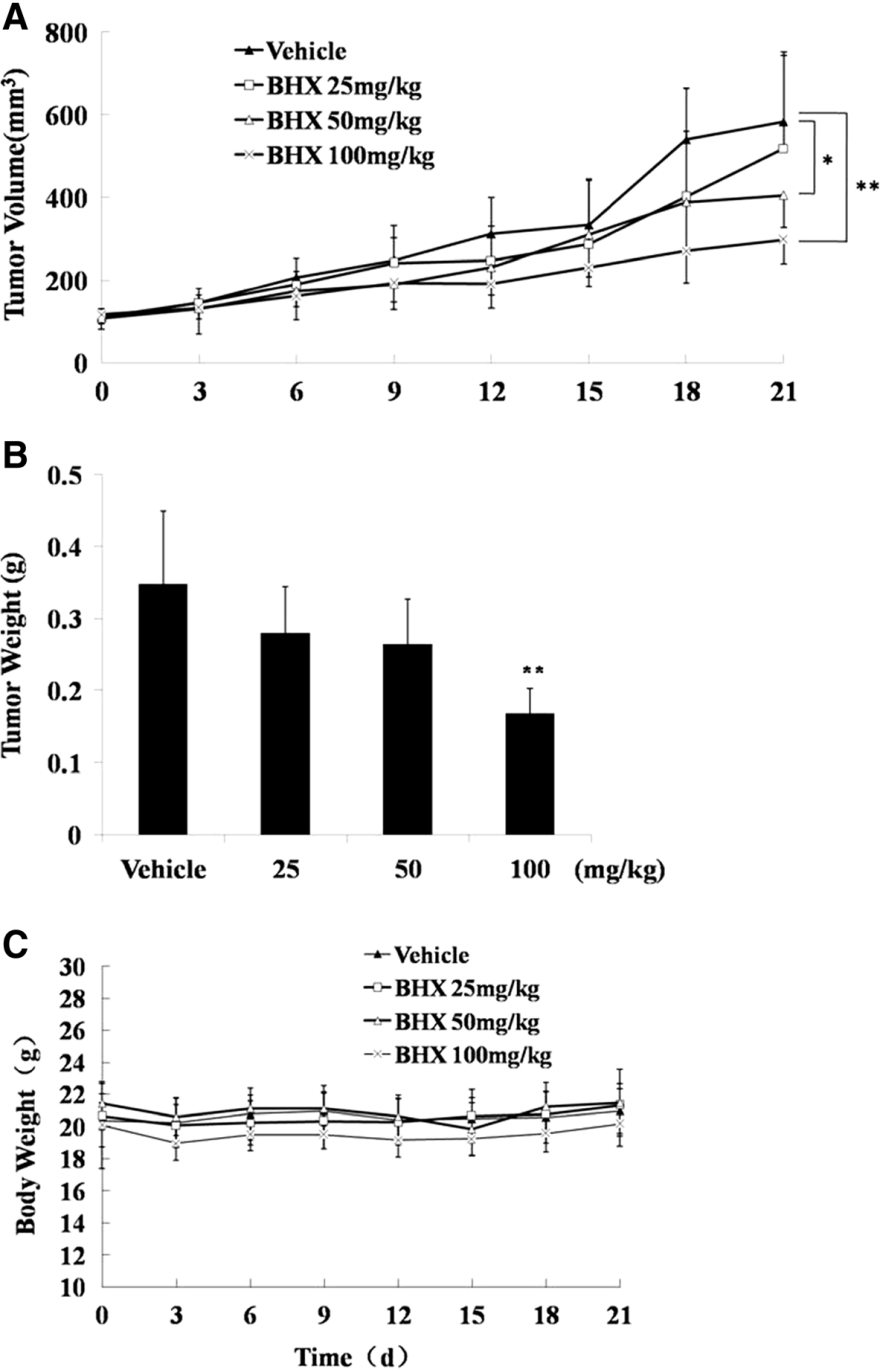
**BHX inhibits A549 cell tumorigenesis in a xenograft model.** Tumor-bearing mice were treated intraperitoneally with vehicle (DMSO) or BHX (25 and 50 mg/kg) daily for 21 d. Tumor size **(A)**, tumor weight **(B)**, and body weight **(C)** were measured as described in the Materials and methods section*.* Data are presented as mean ± SD. *P < 0.05, **P < 0.01, significant relative to vehicle control.

We further investigated the inhibitory effect of BHX on A549 cell tumorigenesis *in vivo* using a mouse xenograft model. BALB/c mice were implanted with A549 lung cells (10^7^ cells/mouse) by subcutaneous injection. When the tumor volumes were approximately 100 mm^3^ to 150 mm^3^, the mice were randomized to receive intraperitoneal injections of vehicle or BHX at 25, 50, and 100 mg/kg for 21 consecutive days. Treatment with 100 mg/kg BHX resulted in a significant reduction in tumor volume (*P* < 0.05, Figure 5A) and tumor weight (*P* < 0.05, Figure 5B) compared with the vehicle group. The inhibition rates in the 100 and 50 mg/kg groups were 50.96% and 29.44%, respectively. By contrast, 25 mg/kg BHX had minimal effect and resulted in an inhibition rate of 10.06%. No significant weight loss was observed in treatment groups or the vehicle group (*P* > 0.05), which indicates that BHX-associated toxicity was minimal (Figure 5C).

## Discussion

Aberrant activation of the Wnt/β-catenin pathway is reportedly among the most important signal transduction pathways in the development and progression of many cancers. Thus, the inhibition of the Wnt/β-catenin signaling pathway has drawn significant interest among cancer researchers. Several molecules have been described to inhibit Wnt signaling in cells. These molecules may act at different steps in the Wnt/β-catenin signal transduction pathway, such as β-catenin/TCF complex, axin2 stability, CREB-binding protein or porcupine enzyme, and antibodies against Wnt proteins. Other inhibitors have been suggested to inhibit Wnt/β-catenin signaling indirectly, such as non-steroid anti-inflammatory drugs and the tyrosine kinase inhibitor (Gleevac) [34]. Several compounds have been tested up to phase I/II clinical trials, but their clinical efficacy and safety have not been established.

In this study, we synthesized a novel small molecule BHX. To confirm the regiochemistry of the cycloaddition for Δ2-pyrazoline **3**, a single crystal for compound 3 was obtained and the absolute configuration was determined by X-ray structural analysis.

However, the X-ray result differed from that in a previous report [22], which indicated that the absolute stereochemistry contradicted our findings. We proposed that cycloaddition could occur from both sides of diphenyl nitrilimine, and that bond formation was favored by the π,π-orbital bond overlap. The transition state showed no stereoselectivity, thus, the Barluenga group, (4R,5S)-(ethyl 1,3,4-triphenyl-4,5-dihydro-1*H*-pyrazole-5-carboxylate, was obtained [35].

Then, we determined that BHX downregulated β-catenin and inhibited cancer cell tumorigenesis *in vitro* and *in vivo*. Our results show that BHX inhibited the growth of cancer cell lines by inducing G1 arrest. The mechanisms underlying the inhibition of Wnt/β-catenin signaling to cause cell cycle arrest remain unclear, but the involvement of Wnt target genes c-myc and cyclin D1 in the G1-S transition may provide an answer [36-38]. Wnt signaling also requires the regulation of SKP2, which is the F-box subunit of the ubiquitinligase complex SCF^SKP2^/p27 degradation pathway, for Wnt signaling-mediated G1-S transition in human urinary bladder cancer cells [39]. BHX possibly has inhibitory effects on the transcriptional activities of target genes c-myc, cyclin D1, and SKP2, which can result in cell cycle arrest during the G1 phase.

Our results also show that BHX induced the increase in E-cadherin protein levels in A549 and MGC803 cells. E-cadherin, a prototypic member of the cadherin single-pass transmembrane glycoprotein family, regulates cell adhesion in epithelial cells in a Ca^2+^-dependent manner [40]. E-cadherin expression level is inversely correlated with tumorigenesis [41,42]. The loss of E-cadherin expression at cell–cell contact is consistently observed at sites of epithelial-mesenchymal transition during tumor development and progression [43]. The Wnt target gene Snail has been suggested to function as a potent repressor of E-cadherin expression [44]. The mechanism of BHX-induced E-cadherin expression is possibly related to the inhibition of the transcriptional activity of Snail.

## Conclusions

In this study, BHX was synthesized, and the absolute configuration of its precursor was determined. This compound showed low nanomolar IC_50_ values and high inhibition rate of nude mice xenografts. Thus, BHX represents a new lead structure for the development of pharmacotherapies to treat human cancers.

## Materials and methods

### Instrument and reagents

Nuclear magnetic resonance (NMR) spectra were obtained on a Bruker spectrometer (AV400, 400 MHz). Chemical shifts (*δ*) are reported in parts per million relative to residual undeuterated solvent (internal reference). The following abbreviations were used to explain the multiplicities: s = singlet, d = doublet, t = triplet, dd = doublet of doublets, m = multiplet, and b = broad. CellTiter 96 cell proliferation assay kit (MTS) was purchased from Promega (Madison, WI, USA). Rabbit anti-human β-catenin polyclonal antibody was purchased from Cell Signaling Technology (Beverly, MA, USA). Rabbit anti-human E-cadherin polyclonal antibody was purchased from Santa Cruz Biotechnology (Santa Cruz, CA, USA). Annexin V-FITC apoptosis detection kit (APO-Direct) was purchased from BD Biosciences (Franklin Lakes, NJ, USA).

### Cells and animals

A549, HT29, and MGC803 were obtained from ATCC (Rockville, MD, USA). A549 and MGC803 cells were cultured in RPMI 1640 culture medium supplemented with 10% fetal bovine serum (FBS, HyClone) at 37°C with 5% CO_2_. HT29 cells were cultured in McCoy’s 5A medium with 10% FBS at 37°C with 5% CO_2_. MCF-10A cells were cultured in Dulbecco’s modified Eagle’s medium (DMEM)/F12 culture medium supplemented with 5% equineserum (HyClone), 20 ng/mL epidermal growth factor, 0.28 U/mL insulin, 500 ng/mL hydrocortisone, and 100 ng/mL cholera toxin. Female BALB/c mice (five to six weeks old) were purchased from the Academy of Military Medical Sciences (Beijing, China). The animals were maintained under the following standardized, environmental conditions: 22°C to 28°C, 60% to 70% relative humidity, 12 h dark/light cycle, and water *ad libitum*.

### X-ray crystallography

Single crystals with suitable dimensions of 0.38 mm × 0.32 mm × 0.15 mm were mounted on glass fibers. Data collection was performed on Brucker SMART Apex CCD diffractometer by ω scan technique using graphite-monochromated Mo-K*α*radiation (*λ* = 0.71073 Å) at 293 K. Compound **3** was crystallized in the triclinic system, space group *P*-1 with *a* = 11.7328(4) Å, *b* = 12.7965(4) Å, *c* = 15.5339(5) Å, *α* = 71.355(2)°, *β* = 89.983(2)°, *γ* = 68.099(2)°, and *V* = 2031.50(11) Å^3^. A total of 31,926 reflections were collected from the θ range of 1.89 to 24.52, and 6750 (R_int_ = 0.0315 [I > 2(I)])/5079 were unique and observed. Empirical absorption corrections were performed using SADABS. The structures were solved by direct methods (SHELXS-97) and refined by full-matrix least squares on F^2^ with weight scheme *w* = 1/[*σ*^2^(*F*_*o*_^2^) + (0.2000*P*)^2^ + 0.0000*P*] (where P = (Fo^2^ + 2Fc^2^)/3) using the SHELXL-97 program suite. All of the non-hydrogen atoms were refined anisotropically, whereas all of the hydrogen atoms were added theoretically. Crystallographic data are summarized in Table 1.

**Table 1:** Crystallographic data and structure refinement for 3

**Formula**	**C**_**24**_**H**_**22**_**N**_**2**_**O**_**2**_
FW	370.44
Crystal system, Space group	Triclinic, *P-1*
*T*, K	293 (2)
*a* (Å)	11.7328 (4)
*b* (Å)	12.7965 (4)
*c* (Å)	15.5339 (5)
**A** (°)	71.355 (2)
*β* (°)	89.983 (2)
*γ* (°)	68.099 (2)
V, Å^3^	2031.50 (11)
*Z*	2
*D*_Calc_, Mg/m^3^	1.211
*μ*, mm^-1^	0.078
*θ* range, °	1.89~24.52
Ranges of indices	−13 ≤ h ≤ 13, -14 ≤ k ≤14, -18 ≤ l ≤18
Total reflections/unique (*R*_*int*_)/observed	31926/6750 (0.0315)/5079
Data/restraints/parameters	6750/0/505
Goodness of fit on *F*^2^	1.094
(*∆ /σ*)_max_	0.004
∆*ρ*, e · Å^-3^ × 10^-3^	0.310, -0.338
*R*^*a*^, *wR*^*b*^	0.0659, 0.0863

***a***,* R* = Σ║*F*_*o*_│-│*F*_*c*_║/∑│*F*_*o*_│; ***b***, *wR* = {[Σ*w*(*F*_*o*_^2^-*F*_*c*_^2^)^2^]/[Σ*w*(*F*_*o*_^2^)^2^]}^1/2^; *w* = 1/[*σ*^2^(*F*_*o*_^2^) + (0.2000*P*)^2^ + 0.0000*P*], where *P* = (*F*_*o*_^2^ + 2*F*_*c*_^2^)/3.

### (*E*)-1-benzylidene-2-phenylhydrazine, 1

Benzaldehyde (306.0 g, 2.9 mol) was slowly added to a mixture of phenylhydrazine (312.0 g, 2.9 mol) and glacial acetic acid (3.1 L). The resulting mixture was stirred at room temperature for 1 h and filtered. The precipitate was washed with acetic acid and H_2_O, and then dried to obtain compound **1** as a white solid (560.0 g, 98.9%). ^1^H-NMR (400 MHz, CDCl_3_) *δ*: 7.65 (m, 4H), 7.36 (m, 2H), 7.27 (m, 3H), 7.11 (m, 2H), and 6.87 (m, 1H).

### Ethyl 1,3,4-triphenyl-4,5-dihydro-1*H*-pyrazole-5-carboxylate, 3

A mixture of ethyl cinnamate (19.3 g, 109.5 mmol), chloramine-T trihydrate (48.9 g, 173.6 mmol), and compound **1** (31.9 g, 162.5 mmol) was dissolved in methanol (71.8 mL) under dim light. The resulting solution was stirred under reflux overnight. After cooling to room temperature, the reaction mixture was poured into a mixture of ethyl acetate and hexane (2.0 L, 1:1) and filtered. The filtrate was concentrated to yield the crude product as a brown oil, which was then purified by column chromatography (Hex: EtOAc, 100:1) to produce compound **2** (4.2 g, 10.4%) as a white powder. ^1^H-NMR (400 MHz, CDCl_3_)*δ*: 7.65 (m, 2H), 7.72 (m, 10H), 7.14 (m, 2H), 6.88 (m, 1H), 4.79 (d, 1H, *J* = 4.3 Hz), 4.68 (d, 1H, *J* = 4.3 Hz), 4.22 (m, 2H), and 1.21 (s, 3H, *J* = 7.1 Hz).

### 1,3,4-Triphenyl-4,5-dihydro-1*H*-pyrazole-5-carboxylic acid, 4

LiOH · H_2_O (0.66 g, 15.9 mmol) was added to a mixture of compound 2 (3.0 g, 8.1 mmol), tetrahydrofuran (THF) (18.0 mL), MeOH (6.0 mL), and H_2_O (6.0 mL). The resulting solution was stirred for 1 h under room temperature. The reaction mixture was concentrated, dissolved in ethyl acetate (40.0 mL), and then respectively washed with 5% aqueous HCl (3 × 20 mL) and brine (3 × 20 mL). The resulting residue was dried over Na_2_SO_4_ and concentrated to produce compound **3** (2.0 g, 72%) as a yellow solid. ^1^H-NMR (400 MHz, DMSO-d^6^)*δ*: 13.32 (s, 1H), 7.70 (d, 2H, *J* = 7.1 Hz), 7.28 (m, 10H), 7.11(d, 2H, *J* = 7.9 Hz), 6.83(t, 1H, *J* = 7.2 Hz), 5.11 (d, 1H, *J* = 3.6 Hz), and 4.68 (d, 1H, *J* = 3.7 Hz).

### N-(4-hydroxybenzyl)-1,3,4-triphenyl-4,5-dihydro-1*H*-pyrazole-5-carboxamide, 5

N-(3-Dimethylaminopropyl)-N’-ethylcarbodiimide hydrochloride (9.0 g, 45.3 mmol), 1-hydroxybenzotriazole (6.1 g, 45.3 mmol), and 4-(aminomethyl)phenol were added to a mixture of compound **3** (14.1 g, 41.1 mmol) in THF (140.0 mL). The resulting solution was stirred overnight under room temperature, and then concentrated to yield the crude product. The pure product (10.4 g, 56.5%) was obtained by column chromatography (Hex: EtOAc, 5:1) as a pale yellow solid. ^1^H-NMR (400 MHz, DMSO-d^6^)*δ*: 9.30 (s, 1H), 8.83 (t, 1H, *J* = 5.8 Hz), 7.67 (d, 2H, *J* = 7.1 Hz), 7.28 (m, 10H), 7.07 (m, 4H), 6.82 (t, 1H, *J* = 7.3 Hz), 6.70 (d, 2H, *J* = 8.8 Hz), 4.93 (d, 1H, *J* = 4.5 Hz), 4.71 (d, 1H, *J* = 4.5 H*z*), and 4.21 (d, 2H, *J* = 5.6 Hz).

### Cell proliferation assay

For 3-(4,5-dimethylthiazol-2-yl)-5-(3-carboxymethoxyphenyl)-2-(4-sulfophenyl)-2H-tetrazolium salt (MTS) cell proliferation assays, A549, MGC803, and HT29 cells were seeded in 96-well culture plates at a density of 500 cells to 1,000 cells per well. After 24 h, BHX or dimethyl sulfoxide (DMSO) was added to the cells at concentrations between 1.25 and 40 μmol/L. Cell proliferation was determined after 72 husing CellTiter 96 AQueous One Solution (Promega). Absorbance was recorded at 490 nm using a microplate reader.

### Apoptosis assays and cell cycle analysis

Cells were seeded at approximately 50% confluence in six-well cell culture plates. BHX (5 μmol/L) or DMSO was added after 24 h, and the cells were collected after 72 h of incubation. Cell apoptosis was measured by Annexin V-FITC/propidium iodide (PI) staining. Cells were considered early apoptotic if Annexin V was positive and PI was negative, and late apoptotic if both Annexin V and PI were positive. For cell cycle analysis, cells were rinsed in PBS, fixed in 95% ethanol, pelleted by centrifugation, and resuspended in RNase A at 50 μg/mL. After incubation at 37°C for 1 h, 50 g/mL PI was added. DNA content was determined by flow cytometry, and the proportion of cells in each cell cycle phase was determined using ModFit software.

### Western blot analysis

Whole cell extracts were lysed in lysis buffer with the following components: 240 mmol/L Tris (pH 6.8), 2% sodium dodecyl sulfate (SDS), 0.5% glycerol, 5 mmol/L EDTA, 1 mg/L aprotinin, 1 mg/L leupeptin, 1 mg/L peptatin A, 10 mmol/L β-glycerol phosphate, 1 mmol/L Na_3_VO_4_, and 1 mmol/L phenylmethylsulfonyl fluoride. Lysates were then spun at 12,000 rpm for 10 min to remove insoluble materials. The protein concentration was measured by BCA protein assay (Pierce). Total proteins were fractionated using SDS–PAGE and transferred to a polyvinylidenefluoride membrane; blotted with antibodies against β-catenin, E-cadherin, and β-actin; and detected by enhanced chemiluminescence (Amersham Biosciences).

### Xenograft studies

Female BALB/c mice, weighing 17 g to 23 g, were implanted subcutaneously with 1 × 10^7^ A549 cells. Tumor sizes were assessed using the two largest perpendicular axes. Tumor volume was calculated using the formula V = (a × b^2^)/2, where *a* is length and *b* is width. When tumor volumes reached 100 mm^3^ to 150 mm^3^, the mice were randomized to drug-treated or vehicle groups (six mice per group). BHX or vehicle control was administered by daily intraperitoneal injection at dose levels of 25, 50, and 100 mg/kg body weight for 21 consecutive days. Tumor growth was monitored every 3 d using a Vernier caliper. Tumor-bearing mice were assessed for weight loss every 3 d. Tumors were removed from mice 21 d after vehicle or BHX treatment. Inhibition rates (antitumor effects) are expressed as T/C% (treatment groups versus control) by dividing the tumor volumes from treatment groups with the control group and then multiplying the quotient by 100. All animal experiments were conducted in accordance with the Animal Care Committee guidelines of Tianjin Medical University Cancer Institute and Hospital.

### Statistical analysis

All values are expressed as mean ± SD. Crude data were analyzed using SPSS 16.0 statistical software. The statistical significance of differential findings between experimental groups and control was determined by Student’s *t*-test or Wilcoxon’s test. *P* values < 0.05 were considered statistically significant.

## Competing interests

The authors declare that they have no potential competing interest with respect to the research, authorship and/or publication of this article.

## Authors’ contributions

ZZ, JW, JS, YC, GY and WC acquired and analyzed data as; performed the statistical analysis; drafted the manuscript. ZY and YD conceived and designed the study; revised and approved the manuscript. All of the authors read and approved the final manuscript.

## Supplementary Material

Additional file 1: Figure S1 BHX dramatically downregulates β-catenin/TCF-dependent transcriptional activity. To measure Wnt signaling, cells at a density of 5 × 10^3^ per well were seeded into 24-well plates before transfection. TOPflash or FOPflash plasmids were co-transfected with PRL-TK plasmid (internal control). Luciferase activity was measured by Dual-Glo Luciferase Assay System after cells were cultured for 18 h. The ratio between firefly luciferase activity (TOPflash/FOPflash) and renilla activity (internal control) was used for TCF/LEF transcription activity. Data represent mean ± SD from three independent experiments. Numerous small molecules were screened for their ability to inhibit Wnt pathway downstream transcriptional activity using the TOP/FOP reporter assay. BHX was observed to reduce β-catenin/TCF-dependent transcriptional activity by 67%. 1: DMSO; 9: BHX. Click here for file

Additional file 2: Figure S2 Effect of BHX on cell growth of MCF-10A or A549. MCF-10A and A549 were plated in triplicate. After 24 h, the cells were treated with DMSO or 3 μmol/L BHX. Viable cell numbers were counted daily for 4 d by trypan blue exclusion. Results are presented as percentage of control. Data are presented as mean ± SD from three independent experiments. Click here for file
