# Supplementary figures and images for: Synthesis, characterization, and evaluation of a novel inhibitor of WNT/β-catenin signaling pathway
Source: Mol Cancer. 2013 Oct 7;12:116. doi: 10.1186/1476-4598-12-116 (PMC3852836; doi:10.1186/1476-4598-12-116)

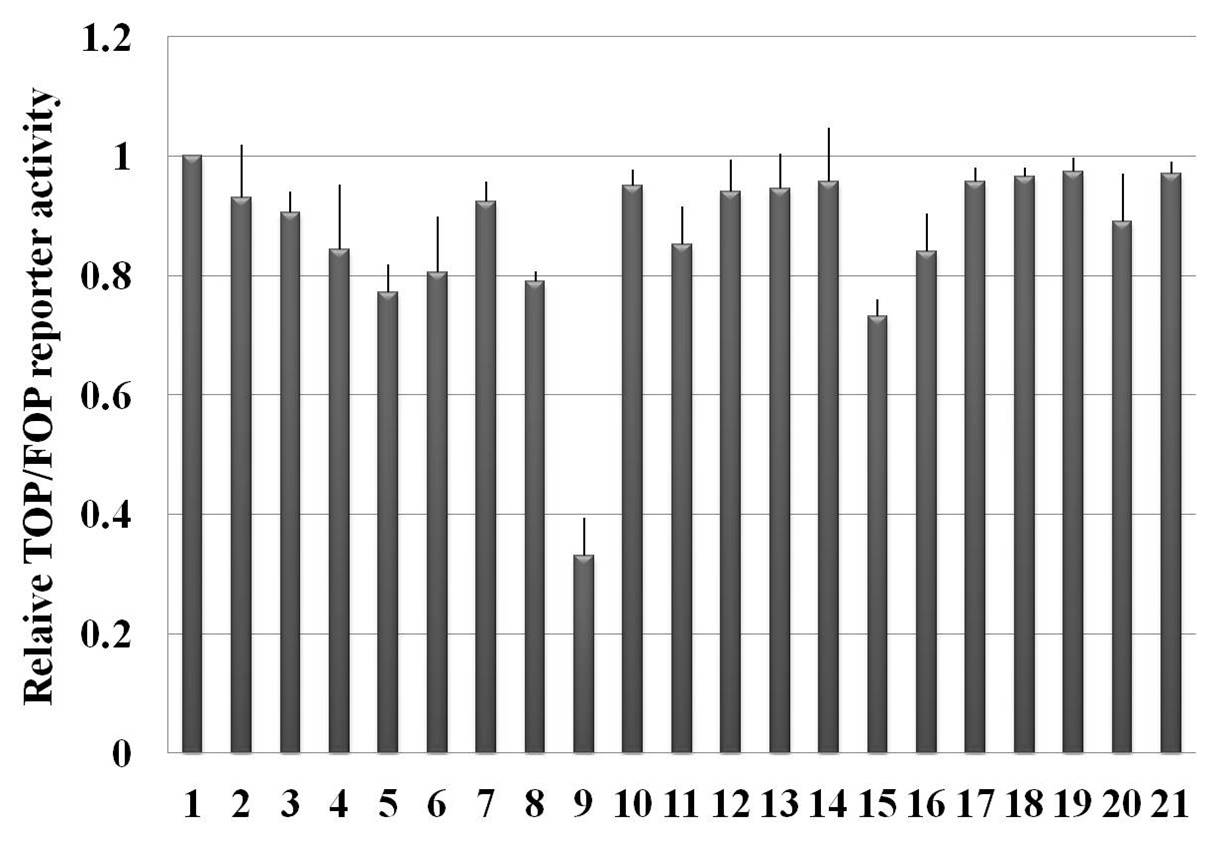

Supplement: Additional file 1: Figure S1 — BHX dramatically downregulates β-catenin/TCF-dependent transcriptional activity. To measure Wnt signaling, cells at a density of 5 × 10^3^ per well were seeded into 24-well plates before transfection. TOPflash or FOPflash plasmids were co-transfected with PRL-TK plasmid (internal control). Luciferase activity was measured by Dual-Glo Luciferase Assay System after cells were cultured for 18 h. The ratio between firefly luciferase activity (TOPflash/FOPflash) and renilla activity (internal control) was used for TCF/LEF transcription activity. Data represent mean ± SD from three independent experiments. Numerous small molecules were screened for their ability to inhibit Wnt pathway downstream transcriptional activity using the TOP/FOP reporter assay. BHX was observed to reduce β-catenin/TCF-dependent transcriptional activity by 67%. 1: DMSO; 9: BHX. [file 1476-4598-12-116-S1.jpeg]

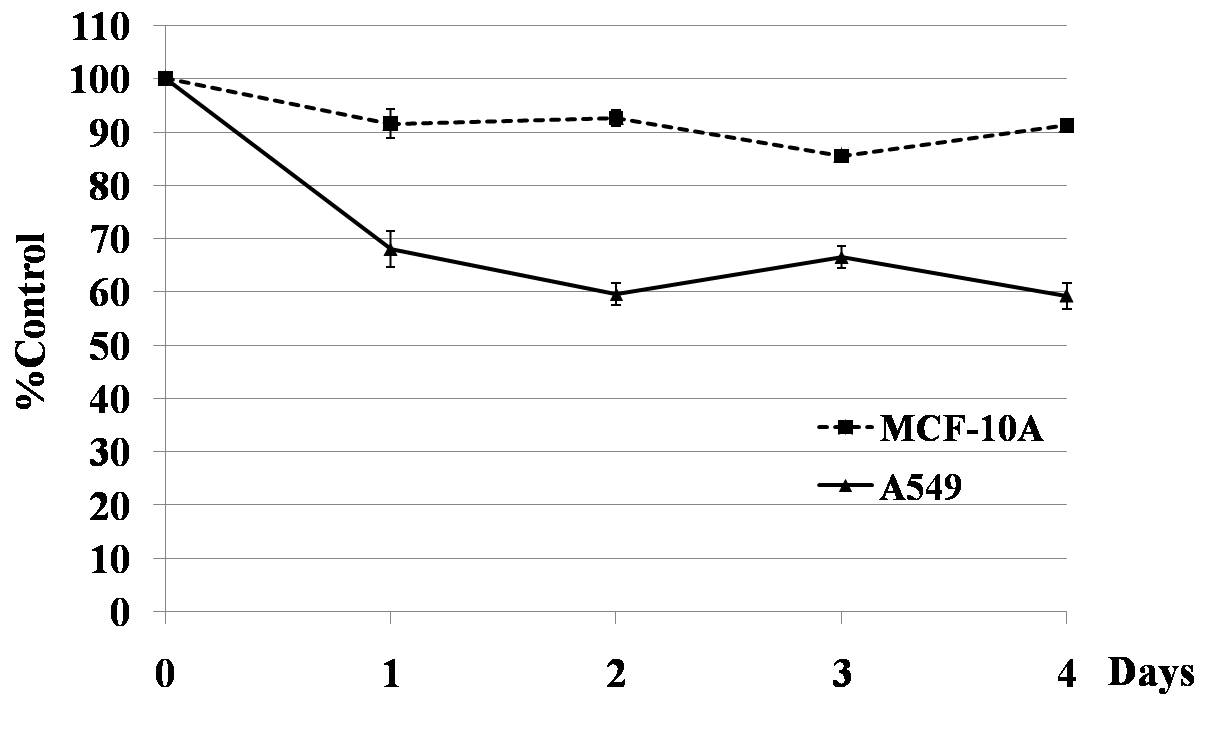

Supplement: Additional file 2: Figure S2 — Effect of BHX on cell growth of MCF-10A or A549. MCF-10A and A549 were plated in triplicate. After 24 h, the cells were treated with DMSO or 3 μmol/L BHX. Viable cell numbers were counted daily for 4 d by trypan blue exclusion. Results are presented as percentage of control. Data are presented as mean ± SD from three independent experiments. [file 1476-4598-12-116-S2.jpeg]
